# Metalloproteinases and Their Tissue Inhibitors in Comparison between Different Chronic Pneumopathies in the Horse

**DOI:** 10.1155/2015/569512

**Published:** 2015-12-03

**Authors:** Ann Kristin Barton, Tarek Shety, Angelika Bondzio, Ralf Einspanier, Heidrun Gehlen

**Affiliations:** ^1^Equine Clinic, Veterinary Faculty, Freie Universitaet Berlin, Oertzenweg 19b, 14163 Berlin, Germany; ^2^Institute of Veterinary Biochemistry, Veterinary Faculty, Freie Universitaet Berlin, Oertzenweg 19b, 14163 Berlin, Germany

## Abstract

In chronic respiratory disease, matrix metalloproteinases (MMPs) contribute to pathological tissue destruction when expressed in excess, while tissue inhibitors of metalloproteinases (TIMPs) counteract MMPs with overexpression leading to fibrosis formation. They may be out of balance in equine pneumopathies and serve as biomarkers of pulmonary inflammation. We hypothesized that MMPs and TIMPs correlate to clinical findings and bronchoalveolar lavage fluid cytology in different equine chronic pneumopathies. Using a scoring system, 61 horses were classified controls as free of respiratory disease (*n* = 15), recurrent airway obstruction (RAO, *n* = 17), inflammatory airway disease (IAD, *n* = 18), or chronic interstitial pneumopathy (CIP, *n* = 11). Zymography and equine MMP and TIMP assays were used to detect MMP-2, MMP-8, MMP-9 as well as TIMP-1, and TIMP-2 in BALF supernatant. MMP-2, TIMP-1, and TIMP-2 concentrations were significantly increased in RAO and IAD compared to controls. MMP-9 concentration and MMP-8 activity evaluated by fluorimetry were significantly increased in RAO, IAD, and CIP. These results were confirmed by zymography for MMP-2 and MMP-9 activity in 52 horses. In conclusion, MMPs and TIMPs correlate well with clinical and cytologic findings. These findings support the usefulness of MMPs, TIMPs, and their ratios to evaluate the severity of respiratory disease and may help to identify subclinical cases.

## 1. Introduction

The extracellular matrix (ECM) represents the scaffold that supports the alveolar wall and has a major impact on lung architecture, homeostasis, and function. The pulmonary ECM underlays a continuous turnover; a dynamic equilibrium between synthesis and degradation of the ECM is maintained for physiological balance. This balance is controlled by synthesis and deposition of ECM components, proteolytic degradation of ECM by matrix metalloproteinases (MMPs), and inhibition of MMP activity by specific tissue inhibitors of matrix metalloproteinases (TIMPs) [1–3]. In health, MMPs degrade the ECM to allow normal tissue repair, but in chronic inflammation they contribute to pathological tissue destruction when expressed in excess [4]. Thus, it has been suggested that MMPs can either protect against or contribute to pathology in inflammatory processes by exacerbation of aberrant lung remodeling [5–7]. ECM degradation results in destruction of interstitial collagen and release of degraded collagen fragments, which results in neutrophil influx with the production of chemoattractants [8]. In chronic respiratory disease, remodeling results in decreasing airway lumen, increased smooth muscle mass, peribronchial fibrosis, epithelial cell hyperplasia, and impaired airway function [9–11]. Regulation of remodeling may be a key for developing new therapeutics and disease management [2].

Matrix metalloproteinases (MMPs) were first described over 50 years ago by Gross and Lapiere [12]. Collagenolytic MMP-8 was increased in tracheal epithelium lining fluid (TELF) of RAO affected horses [13]. Immunoreactivity of collagenases MMP-8 and MMP-13 was significantly increased in TELF of horses with RAO, compared to healthy horses, and was positively correlated with the amount of degradation of type-I collagen [14].

Markedly increased elastolytic activity in TELF was also found in RAO, suggesting participation of elastases (MMP-2, MMP-3, MMP-7, MMP-9, MMP-10, and MMP-12) [15]. Other authors found no difference in pro-MMP-2 compared to healthy horses and suggested that MMP-2 may represent a housekeeping proteinase involved with normal tissue remodeling [16]. Previously it has been described that the molecular weight of pro-MMP-2 is 65–75 kDa and that of lower molecular weight gelatinolytic species is below 50 kDa [17]. In horses, MMP-9 is found elevated in RAO affected horses. In TELF and BALF MMP-9-related gelatinase-activity was represented by 5 bands: high molecular weight gelatinase complex (above 110 kDa), pro-MMP-9 (90–110 kDa), and active MMP-9 (75–85 kDa) [17]. In tracheal aspirates of RAO affected horses, mainly high molecular weight bands (150–210 kDa) and 90–110 kDa bands were found in symptomatic disease phases compared to healthy horses [16]. MMP-9 represents the largest and complex member of MMPs that is present in low quantities in the healthy adult lung but much more abundant in several lung diseases, including asthma, idiopathic pulmonary fibrosis, and RAO [18]. BALF gelatinolytic MMP activity in RAO affected horses increases as early as 5 hours after natural challenge and correlates with the BALF neutrophil counts [18, 19].

Tissue inhibitors of metalloproteinases are specific inhibitors of MMPs that bind to MMPs and inhibit their enzymatic activity. Four TIMPs have been identified including TIMP-1, TIMP-2, TIMP-3, and TIMP-4 and inhibit all MMPs tested [20, 21]. In human COPD, increased MMP-9 and TIMP-1 concentrations were detected in plasma and BALF [22].

TIMP-1 is the most widely distributed and acts on all active MMPs. A higher concentration of TIMP-1 was found in human BALF of asthmatic patients compared to healthy controls; thus it might be a better marker for mild asthma [23]. Also, high levels of TIMP-1 are associated with increased airway fibrosis. In addition, the molar concentration of TIMP-1 often exceeds the concentrations of MMP-9 and other MMPs [24]. These findings suggest that although TIMP-1 protects airway tissue from enhanced MMP activity, its increase may also be pathogenic and lead to enhanced airway fibrosis.

TIMP-2 appeared to be effective in preventing ECM damage by inhibition of MMP-2 and related proteolytic activity. Additionally, it serves as a target for therapy as reduced airway inflammation and hyperresponsiveness were observed after the administration of recombinant TIMP-2 in the bronchial tree [25].

In addition to MMP and TIMP concentrations, MMP : TIMP ratios have raised increasing interest in human asthma and COPD. They have been found to be even more sensitive biomarkers than MMPs and TIMPs. To our knowledge, TIMPs and MMP : TIMP ratios in equine pulmonary disease have not been studied so far.

While the role of MMPs in RAO has been studied intensively in the horse, not much is known about other chronic respiratory diseases leading to exercise insufficiency and therefore representing a major economic problem in the horse industry. We hypothesized that MMP-2, MMP-8 and MMP-9 as well as TIMP-1 and TIMP-2 concentrations correlate with clinical and cytologic data and increase in different forms of chronic pneumopathy including RAO, inflammatory airway disease (IAD), and chronic interstitial pneumopathy (CIP). We also suspected that MMP : TIMP might be valuable biomarkers in equine disease.

## 2. Materials and Methods

### 2.1. Preparticipation Examination

A total of 64 horses were examined, of which 15 had no clinical signs or history of respiratory disease and 49 were presented to the clinic with a history of chronic lower airway disease. Sampling of horses affected by respiratory disease was not classified as animal experiments by the State Office of Health and Social Affairs Berlin (LaGeSo); sampling of control horses was approved (reference number L0294/13). The owners gave permission to involve their horses in the study.

The preparticipation examination included anamnesis documentation, clinical examination, exercise test, blood gas analysis, bronchoscopy, BALF cytology, and thoracic radiography. A modified clinical score system including endoscopy results, parameters of gas exchange, and BALF cytology was used, shown in Table 1 [26, 27]. Additionally, results of thoracic radiography were included to classify horses as free of respiratory disease (controls), recurrent airway obstruction in exacerbation (RAO), inflammatory airway disease or RAO in remission (IAD), and chronic interstitial pneumopathy (CIP) or were excluded from the study, if they could not be assigned to these groups. In detail, groups were defined as follows:

- Controls: no history of respiratory disease, clinical score <2, no tracheal secretions, low cellular density and neutrophils ≤ 8% in BALF, AaDO_2_ ≤8 mmHg, and exclusion of acute signs of infection (leukocytosis, fever, and depression).
- RAO: history of recurrent cough or dyspnea, clinical score >6, high amount or viscosity of tracheal secretions, high cellular density and neutrophils ≥25% in BALF, AaDO_2_ >8 mmHg, and exclusion of acute signs of infection (leukocytosis, fever, and depression) according to Robinson [28].
- IAD: history of cough or exercise insufficiency, clinical scores 2–6, low to moderate amount or viscosity of tracheal secretions, increased cellular density and neutrophils ≥8% or mast cells ≥2% or eosinophils ≥0.1% in BALF, and exclusion of acute signs of infection (leukocytosis, fever, and depression) according to Couëtil et al. [29].
- CIP: history of exercise insufficiency, clinical score 2–6, low to moderate amount or viscosity of tracheal secretions, increased cellular density and ratio of macrophages: neutrophils ≥2.5 : 1 in BALF, increased interstitial opacity of thoracic radiographs, and exclusion of acute signs of infection (leukocytosis, fever, and depression) according to Dieckmann et al. [30].

### 2.2. BALF Collection and Processing

During endoscopy, 20 mL of 2% lidocaine (Lidocaine, Bela-Pharm GmbH, Vechta, Germany) was infused around the tracheal bifurcation. The catheter (Silicone Bronchoalveolar Lavage Catheter 300 cm, Smiths Medical ASD, Inc., USA) was wedged into the bronchus by mean of an air balloon. Five hundred milliliters of prewarmed phosphate buffered saline (phosphate buffered saline, Lonza, Verviers, Belgium) was infused as recommended by the International Workshop on Equine Chronic Airway Disease [28] and immediately aspirated.

BALF was divided into 2 portions for cytological and biochemical examination. After centrifugation (Table Top Refrigerated Centrifuge Hermle Z326K, Hermle Labortechnik GmbH, Germany) at 1500 rpm for 10 min at 4°C the cell-free supernatant was stored at −80°C until being assayed. Cytology was performed using Wright-Giemsa staining and counting 500 cells at 500x magnification.

### 2.3. Gelatin Zymography (MMP-2 and MMP-9)

Zymography was performed (gelatin zymogram gels (Life Technologies, USA); electrophoresis device XCell, Novex Experimental Technology, Japan) according to the manufacturer's manual. Human MMP-2 and MMP-9 controls (recombinant human MMP-2, USCN Life Science, Inc., China) were used together with a multicolor broad protein range protein ladder (Spectra Multicolor Broad Range Protein Ladder, Thermo Scientific, Rockford, USA) as control on each gel. Also, a sample of a control horse was applied to each gel to compare the signals to affected horses. Gels were scanned for digital analysis by densitometry using digital image analyzing software (ImageJ v1.47, Wayne Rasband, NIH, USA) for objective quantification of bands and the results were presented based on peak area [31].

### 2.4. ELISA of MMP-2, MMP-9, TIMP-1, and TIMP-2

The ELISAs used in this study were equine specific sandwich enzyme immunoassays for quantification of MMP and TIMP concentrations (equine MMP-2 kit, USCN Life Science, Inc., China; equine MMP-9 kit, USCN Life Science, Inc., China; equine TIMP-1 kit, USCN Life Science, Inc., China). Standards and samples were set up in duplicate according to the manufacturer's protocol. The absorbance was measured with an ELISA microplate reader (equine TIMP-2 kit, USCN Life Science, Inc., China) at 450 nm immediately. Calculation of the unknown sample concentration was made by inverted standard curve using the* Excel* software program.

### 2.5. Fluorimetry MMP-8 Assay

The MMP-8 assay (ELISA microplate reader, BioRad Laboratories, USA) was performed according to the manufacturer's protocol. Negative controls containing assay buffer and positive controls using recombinant human purified MMP-8 were included.

### 2.6. Statistical Analysis

Data were statistically analyzed using SPSS (Sensolyte 520 MMP-8 Assay Kit, Anaspec, Inc., Fermont, USA) and expressed as mean ± standard deviation (SD). The data were tested for normal distribution using the Shapiro Wilks Test. While some data were found to be normally distributed, other was not, so we preferred nonparametric tests for the whole data. The level of significance was set at *p* < 0.05.

Kruskal Wallis *H* test was used to compare between controls and different disease groups followed by post hoc testing using Mann-Whitney *U* Test for 2-group comparison to determine intergroup differences.

Spearman rank correlation coefficients were calculated between clinical examination scores and blood gas scores and between these variables and the total examination score, neutrophil percentages, MMP-2, MMP-9 concentrations, and MMP-8 activity. The Spearman correlation coefficients were interpreted using the scale provided by Salkind, where the values between 0.8 and 1.0, 0.6 and 0.8, 0.4 and 0.6, 0.2 and 0.4, and 0.0 and 0.2 were defined as very strong, strong, moderate, weak, and very weak or no relationship, respectively [32]. The same was performed for calculated MMP : TIMP ratios.

## 3. Results

According to the results of the clinical examination, the 64 horses (35 geldings, 29 mares, aged 12.74 ± 5.25 years, BDW 473.79 ± 5.26 kg) presented for participation in this study were classified as follows: 15 horses (23.4%) were classified as free of respiratory disease (controls), 17 (26.6%) as RAO in exacerbation, 18 (28.1%) as RAO in remission or IAD, and 11 (17.2%) as CIP and 3 horses (4.7%) suffered from acute respiratory infections and were excluded. The overall results of the clinical examinations are presented in Table 2.

### 3.1. Endoscopy

The results of the endoscopic examination including the amount and viscosity of secretions and the tracheal bifurcation appearance were scored according to Dieckmann and Deegen [33] and Gerber et al. [34] and included into a modified overall clinical score [26, 27]. The results are presented in Table 3.

### 3.2. Pulmonary Function

The results of arterial blood gas analysis are presented in Table 4. PaO_2_ and AaDO_2_ were significantly increased in RAO but not in other disease groups.

### 3.3. BALF Cytology

Percentages of macrophages, lymphocytes, mast cells, and in particular neutrophils were highly and significantly different between controls and different disease groups (Table 5). BALF neutrophils percentage (reference range 0–8%) was significantly increased in RAO (60.68 ± 21.59%), IAD (15.64 ± 8.19%), and CIP (8.73 ± 5.71%) compared to controls (3.02 ± 2.41%). Also, BALF neutrophils percentage was significantly increased in RAO compared to IAD and CIP.

### 3.4. MMP-2 ELISA

In RAO (5.21 ± 0.77 ng/mL) and IAD (7.67 ± 15.5 ng/mL) highly significant increases in MMP-2 concentrations were found compared to controls (2.49 ± 0.83 ng/mL). MMP-2 was not increased in CIP (2.81 ± 0.34 ng/mL). Horses diagnosed with IAD showed a highly significant increase compared to RAO and CIP.

### 3.5. MMP-9 ELISA

Obvious differences between disease groups were detected. Highly significant increases in MMP-9 concentration were found in RAO (433.34 ± 89.05 pg/mL), IAD (312.06 ± 23.92 pg/mL), and CIP (263.2 ± 23.85 pg/mL) compared to controls (176.29 ± 60.22 pg/mL). In RAO a highly significant increase compared to IAD and CIP was evident. Other intergroup differences were not found significant.

### 3.6. MMP-8 Activity

MMP-8 activity showed significant differences between all groups. In RAO (21,802.03 ± 21,047 RFU), IAD (5,366.17 ± 1,434 RFU), and CIP (3,800.36 ± 403 RFU) significant increases were found compared to controls (3,556.63 ± 176 RFU). Intergroup analysis revealed highly significant increases in MMP-8 activity in RAO compared to IAD and CIP. A significant increase was also found in IAD compared to CIP.

### 3.7. TIMP-1 ELISA

Highly significant increases in TIMP-1 concentrations were found in RAO (328.19 ± 62.83 pg/mL) and IAD (308.92 ± 8.24 pg/mL) compared to controls (117.54 ± 45.62 pg/mL). Intergroup differences were not significant.

### 3.8. TIMP-2 ELISA

TIMP-2 concentrations were also highly significantly increased in groups RAO (27.75 ± 5.08 ng/mL) and IAD (25.42 ± 1.38 ng/mL) compared to controls (18.06 ± 2.37 ng/mL). Intergroup differences were not significant.

### 3.9. MMP-TIMP Ratios

Significant differences were found for RAO in MMP-9/TIMP-2, MMP-8/TIMP-1, and MMP-8/TIMP-2 ratios, for IAD in MMP-2/TIMP-2 and MMP-8/TIMP-1 ratios, and for CIP in the MMP-8/TIMP-1 ratio.

A concluding summary of all MMP- and TIMP-measurements is presented in Table 6 and for MMP/TIMP ratios in Table 7.

### 3.10. Gelatin Zymography

**Figure 1:**
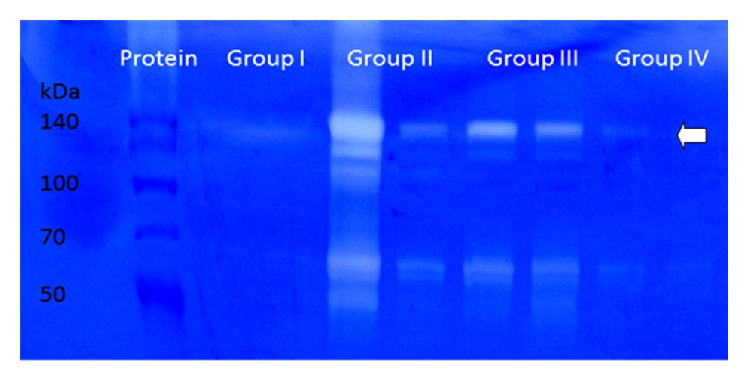
Gelatin zymography of MMP-2 and MMP-9. Examples of healthy controls (group I), RAO (group II), IAD (group III), and CIP (group IV). The 70 kDa bands are representative of pro-MMP-2 and those at 140 kDa are representative of high molecular weight MMP-9 (arrow) as checked in a comparison of protein marker and human MMP-2 and MMP-9 (data not shown).

Gelatin zymography was performed on 52 BALF samples from controls (*n* = 13), RAO (*n* = 17), IAD (*n* = 14), and CIP (*n* = 8) affected horses. Gelatinolytic activity bands were detected at about 70 kDa for MMP-2 (pro-MMP-2) and at 100 and 140 kDa for MMP-9, respectively (pro-MMP-9 and high molecular weight forms). An example of a zymographic gel is shown in Figure 1.

Based on peak areas, high molecular weight bands of MMP-9 showed significant differences between groups. In RAO (10,967.31 ± 9,530.07) highly significant increases in peak areas were found compared to controls (619.29 ± 996.32) and also showed a highly significant increase compared to IAD (1,832.16 ± 2,111.29) and CIP (864.06 ± 767.93). Other intergroup differences were not found significant.

Digital analysis for MMP-2 was based on bands of the gelatinolytic pro-MMP-2. Bands showed highly significant differences between RAO (17,288.53 ± 8,927.59) and IAD (3,530.94 ± 2,894.15) compared to controls (1,114.76 ± 672.72). Peak areas were significantly increased in RAO compared to IAD and CIP (2,799.45 ± 2,592.28, Figure 1). Other intergroup differences were not significant.

### 3.11. Correlation of MMPs, TIMPs and Clinical Score

The total clinical examination score showed a positive correlation with the concentrations of MMP-2 (*r* = 0.75), MMP-8 (*r* = 0.77), MMP-9 (*r* = 0.79), TIMP-1 (0.65), TIMP-2 (0.72), MMP-8 : TIMP-1 (0.76), and MMP-8 : TIMP-2 ratio (0.90). Also, a positive correlation was found with the MMP-2 (*r* = 0.80) and MMP-9 (*r* = 0.71) activity measured by gelatin zymography. All of these correlations had a level of significance of *p* < 0.01.

### 3.12. Correlations of MMPs, TIMPs and BALF Cytology

BAL neutrophil percentages showed a positive correlation with the concentrations of MMP-2 (0.77), MMP-8 (0.76), MMP-9 (0.81), TIMP-1 (0.65), TIMP-2 (0.71), MMP-8 : TIMP-1 (0.90), and MMP-8 : TIMP-2 ratio (0.98). Also, a positive correlation was found with the MMP-2 (0.78) and MMP-9 (0.67) activity measured by gelatin zymography. All of these correlations had a level of significance of *p* < 0.01.

## 4. Discussion

In horses, collagenolytic and elastolytic MMPs in pulmonary secretions have been shown to increase during RAO [13–16]. In the present study, increased concentrations were found not only in RAO but also in IAD and chronic interstitial pneumopathies by semiquantitative and quantitative measurements and were highly correlated with the results of the clinical examinations and BALF cytology. TIMP concentrations were also increased in RAO and IAD but not in CIP. Healthy horses seem to have minimal gelatinolytic and collagenolytic activities, as MMP activity is physiologically balanced by TIMPs. An imbalance between MMP expression, activation, and inhibition is associated with tissue destruction in inflammatory lung diseases.

In the present study, MMP-2 and MMP-9 were identified using gelatin zymography as described previously in equine RAO [13, 17]. Human MMP markers served as controls due to unavailability of the purified equine protein. Although densitometry was used for quantification of the bands and the results were calculated based on peak area [31], we also aimed for direct quantitative measurements using equine ELISA kits. Quantification revealed the highest MMP-8 and MMP-9 activities in RAO, but activities in IAD and CIP were also significantly increased compared to controls.

In a study on tracheal epithelium lining fluid, the concentration of autoactive collagenase was approximately 7 times greater in RAO [14]. The authors concluded that collagenases are also involved in airway remodeling during exacerbation. Several studies have shown that MMP-9 is the main MMP present in the airways of RAO-susceptible horses following inhalation of hay dust or its components [19, 35, 36].

In unison with previous studies [15–17], our results of this study support the role of MMP-9 in RAO but also suggest a role of collagenases in equine IAD and CIP. The high positive correlations of MMPs and neutrophil percentages in BALF suggest these cells to be the origin of MMPs, in particular MMP-9, in RAO. Much debate exists on a possible precursor role of IAD for the development of RAO [29]. Increased MMP-8 activity in both groups may further support this theory. In addition, it seems possible that CIP may develop from IAD, which has a high prevalence in young sports horses, while RAO and CIP are more common in older individuals. In our study, we also found a correlation of MMP-8 concentrations and neutrophil percentage in BALF samples. Increased collagenase activity in lungs of humans with emphysema and bronchiectasis is suspected as a result of MMP-8 activity [37, 38]. All immunoreactive forms of MMP-8 detected in TELF samples were also detected in equine neutrophil lysate. Therefore, neutrophil-derived MMP-8 species were suggested to be the cause of the MMP-8 immunoreactivity detected in TELF samples [14]. Immunoreactivity for MMP-8 in TELF from RAO horses was approximately 13 times greater than in controls [14]. The factor between RAO horses and controls is even larger in our study and there were also significant increases in IAD and CIP. Nevertheless, the highest concentrations were measured in RAO with significant differences to the other groups.

The role of MMP-2 is of controversy in the literature. MMP-2 has been considered to be constitutively expressed [14, 35] and therefore its induction in inflammation has rarely been detected. Our results support this theory, as zymography only revealed bands of pro-MMP-2 in most cases. The MMP-2 ELISA also showed the highest values in horses suffering from IAD, which is characterized by a lower grade of inflammation and pulmonary dysfunction compared to RAO in exacerbation. In addition, no MMP-2 increase was found in CIP; therefore a constitutive role for this enzyme remains likely.

In men, increases in TIMP-1 and TIMP-2 have been found in asthma and COPD. Results of the presented study show that the same is true for equine RAO and IAD, but not for CIP. This makes sense, as CIP represents the final stage of interstitial pneumonia, characterized by a low grade of inflammation and organized fibrotic tissue. High correlations of MMPs as well as TIMPs with clinical findings and BALF cytology over all horses substantiate this result. Despite these obviously clear results, MMP : TIMP ratios raised our special interest, as they show a possible disbalance between ECM degradation and repair mechanisms leading to either pulmonary tissue destruction or fibrosis formation in the end. Perhaps the most interesting one was the MMP-8 : TIMP-1 ratio, in which significant differences were found for all pneumopathies studied compared to healthy controls, but in different orientations. While RAO was characterized by predominating collagenolytic activity demonstrated by an increased ratio, IAD/RAO in remission and CIP were characterized by predominating fibrosis formation demonstrated by a decreased ratio. This shows that even forms of equine respiratory disease going along with very slight clinical signs and cytologic findings lead to fibrosis formation affecting prognosis in these patients. Additionally, the MMP-8 : TIMP-1 ratio can identify cases of respiratory disease, in which clinical signs and cytologic findings are almost unremarkable, which is very helpful, when examining horses in remission.

Equine RAO shows features of human asthma and COPD. While the recurrent character of the disease resembles asthma, long-term changes include irreversible remodeling of the bronchial wall leading to decreased gas exchange and pulmonary function as known for COPD. In asthma, the degree of MMP activity can be linked to intensity of the inflammatory processes in the airways; therefore the MMPs/TIMPs balance is widely accepted to have a role in the pathogenesis of airflow limitation and reflect the extent of structural changes in the lung [39–41]. Mediators released by activated parenchymal and inflammatory cells could induce MMPs secretion and activation, increase in MMP-9 activity, and elevated MMP-9/TIMP-1 ratios as demonstrated in mild asthma after allergen challenge in sputum and BALF [23, 42]. Specific allergen challenge is also able to induce changes in MMPs and TIMPs, in particular MMP-9, in occupational asthma [43]. In severe asthma, increased basal levels of MMP-9 were even observed in plasma [44]. No difference was also found for MMP-2, again supporting a constitutive role as discussed earlier.

Equine RAO also shows features of human COPD, in which long-term exposure to cigarette smoke, toxic gases, and particulate matter leads to airflow limitation and pulmonary failure [39]. As RAO the disease is characterized by an excess of extracellular matrix deposition in bronchial walls, known as remodeling and involving many members of the MMP family, chronic cough, and dyscrasia. Increased MMP-1 and MMP-9 levels have been detected in BALF of emphysema patients [38]. COPD patients show increased activities of MMP-2 and MMP-9 in their lung parenchyma [45] and increased gelatinolytic activity linked to MMP-2 and MMP-9 in their sputum [46, 47]. An increase of collagenolytic activity, probably due to elevated levels of MMP-8, was also found [48].

Chronic interstitial pneumopathy is a poorly defined disease in equine medicine. Descriptions in the literature are rare [49]. Dieckmann et al. [30] gave some definition criteria from the examination of 12 affected patients and Venner et al. [50] studied horses after experimental induction of acute interstitial pneumopathy. In the early stage of human lung fibrosis, gelatinolytic activity of MMP-9 seems predominant and probably contributes to disruption of alveolar epithelial basement membrane and enhances fibroblast invasion to alveolar spaces [39], while, in the late stages of the disease, MMP-2 seems to become predominant. The expression of the two gelatinases at different stages of fibrosis suggests that MMP-9 could be rather linked to inflammation-induced tissue remodeling, while MMP-2 may be associated with an impaired tissue remodeling leading to pathological collagen deposition and interstitial fibrosis [41].

A weak point of this study was group definition, as samples were obtained from clinic patients. Although IAD and RAO in remission were planned as two distinct groups, it was not possible to differentiate clearly between these two. Anamnestic information of respiratory distress was often unreliable and the majority of owners did not agree to a natural challenge test. Descriptions of equine CIP are rare in the literature and an international consensus statement is missing, so definition of this group was based on a quite old clinical case series including only 12 horses [30]. Thoracic radiography showing interstitial patterns is not very specific for CIP and may also be found in RAO [51] and IAD [52]. Again, the majority of owners did not agree to lung biopsies. We tried to face these problems by calculating correlations between MMPs and TIMPs with clinical and cytologic parameters over all 61 horses and found significant results for almost all correlations, demonstrating the value of MMPs, TIMPs, and MMP : TIMP ratios as biomarkers independent of diagnosis.

In conclusion, metalloproteinases and their inhibitors, in particular MMP-9 and TIMP-1, are increased in different chronic pneumopathies in the horse and correlate significantly with clinical and cytologic findings. MMPs, TIMPs, and in particular the MMP-8 : TIMP ratios are useful to evaluate the severity and character of respiratory disease and may have prognostic value for equine pneumopathies. Further studies should focus on the balance between MMPs and TIMPs and their progression during disease and possible improvement during therapy.

## Figures and Tables

**Table 1:** Clinical scoring system, modified from Ohnesorge et al. (1998) [26] and Gehlen et al. (2008) [27].

		Score	Max. points
(1) Cough induction	No cough after manual compression of larynx	0	1
	Coughing during manual larynx compression	1	
	Very frequent coughing	1	
	Spontaneous coughing	1	

(2) Dyspnoea at rest	Prolonged expiration	1	3
	Abdominal breathing	1	
	Sinking of the intercostal area	3	
	Nostril flare	3	
	Heaves line	3	
	Anal pumping	3	

(3) Lung percussion	3 fingers	0	2
	Handbreadth	1	
	Damping	2	

(4) Lung auscultation	Rattling	2	2
	Crackle	2	
	Wheezing	2	

(5) Endoscopy	Significantly increased secretions with moderate viscosity	1	2
	Highly increased secretions with high viscosity	2	
	Thickened carina of the trachea	1	

(6) BALF cytology	Neutrophils <8%	0	3
	Neutrophils 8–15%	1	
	Neutrophils 15–25%	2	
	Neutrophils >25%	3	

(7) Arterial blood gas analysis	AaDO_2_: 0–7 mmHg	0	2
	AaDO_2_: 7–14 mmHg	1	
	AaDO_2_: >14 mmHg	2	

**Table 2:** Results of clinical examinations. *∗* shows significant increase to controls at *p* < 0.05 and *∗∗* significant increase in RAO compared to IAD and CIP. RAO: recurrent airway obstruction, IAD: inflammatory airway disease, and CIP: chronic interstitial pneumopathy.

	Controls (*n* = 15)	RAO (*n* = 17)	IAD (*n* = 18)	CIP (*n* = 11)
Endoscopy score	0 ± 0	1.82 ± 0.39^*∗*^	1.17 ± 0.71^*∗*^	0.91 ± 0.7^*∗*^
BAL score	0 ± 0	3 ± 0^*∗*^	1.39 ± 1.04^*∗*^	0.45 ± 0.69
Blood gas score	0.2 ± 0.4	0.76 ± 0.75^*∗*^	0.17 ± 0.38	0.36 ± 0.81
Total exam score	0.27 ± 0.46	8.12 ± 2.23^*∗*,*∗∗*^	3.88 ± 1.41^*∗*^	2.36 ± 2.66^*∗*^

**Table 3:** Results of endoscopic examination. *∗* shows significant increase to controls at *p* < 0.05. RAO: recurrent airway obstruction, IAD: inflammatory airway disease, and CIP: chronic interstitial pneumopathy.

	Amount of secretions	Viscosity of secretions	Tracheal bifurcation
Controls (*n* = 15)	0.47 ± 0.64	0.4 ± 0.51	0.25 ± 0.45
RAO (*n* = 17)	3.5 ± 0.63^*∗*^	3.88 ± 0.5^*∗*^	1.36 ± 1^*∗*^
IAD (*n* = 18)	2 ± 1.33^*∗*^	2.33 ± 1.4^*∗*^	1.33 ± 0.82^*∗*^
CIP (*n* = 11)	2 ± 1.2^*∗*^	1.8 ± 1.2^*∗*^	1.56 ± 0.88^*∗*^

**Table 4:** For arterial blood gas analysis, the results are expressed as mean ± SD. *∗* shows significant difference to controls at *p* < 0.05. RAO: recurrent airway obstruction, IAD: inflammatory airway disease, and CIP: chronic interstitial pneumopathy.

	PaCO_2_ [mmHg]	PaO_2_ [mmHg]	AaDO_2_ [mmHg]
Controls (*n* = 15)	43.87 ± 2.53	101.95 ± 6.18	0.52 ± 1.03
RAO (*n* = 17)	43.63 ± 4.76	87.9 ± 12.15^*∗*^	10.91 ± 9.3^*∗*^
IAD (*n* = 18)	43.75 ± 3.02	94.98 ± 7.38	5.17 ± 8.03
CIP (*n* = 11)	44.19 ± 3.24	94.23 ± 9.4	5.55 ± 8.5

**Table 5:** For BAL cytology, the results of cell percentages are expressed as mean ± SD. *∗* shows significant differences to controls at *p* < 0.05, *∗∗* significant increase in RAO compared to IAD and CIP, and *∗∗∗* significant decrease in RAO compared to IAD and CIP. RAO: recurrent airway obstruction, IAD: inflammatory airway disease, and CIP: chronic interstitial pneumopathy.

	Macrophages [%]	Lymphocytes [%]	Neutrophils [%]	Eosinophils [%]	Mast cells [%]
Controls (*n* = 15)	56.48 ± 4.75	38.15 ± 6.41	3.02 ± 2.41	0.13 ± 0.27	2.22 ± 2.06
RAO (*n* = 17)	19.64 ± 12.07^*∗*,*∗∗∗*^	18.66 ± 12.16^*∗*^	60.68 ± 21.59^*∗*,*∗∗*^	0.27 ± 0.35	1.16 ± 1.22
IAD (*n* = 18)	43.78 ± 12.98^*∗*^	34.63 ± 13.65	15.64 ± 8.19^*∗*^	1.95 ± 3.9	3.8 ± 3.21
CIP (*n* = 11)	50.83 ± 15.46	34.47 ± 11.87	8.73 ± 5.71^*∗*^	0.95 ± 0.92	5.03 ± 4.34

**Table 6:** MMP-2, MMP-9, TIMP-1, TIMP-2 ELISA, and MMP-8 fluorimetry measurements. The results are expressed as mean ± SD. *∗* shows significant increases to controls at *p* < 0.05, *∗∗* significant increase in IAD compared to RAO and CIP, and *∗∗∗* significant increase in RAO compared to IAD and CIP. RAO: recurrent airway obstruction, IAD: inflammatory airway disease, and CIP: chronic interstitial pneumopathy.

	MMP-2 [ng/mL]	MMP-9 [pg/mL]	MMP-8 [RFU]	TIMP-1 [pg/mL]	TIMP-2 [ng/mL]
Controls (*n* = 15)	2.49 ± 0.83	176.29 ± 60.22	3,556.63 ± 176	117.54 ± 45.62	18.06 ± 2.37
RAO (*n* = 17)	5.21 ± 0.77^*∗*^	433.34 ± 89.05^*∗*,*∗∗∗*^	21,802.03 ± 21,047^*∗*,*∗∗∗*^	328.19 ± 62.83^*∗*^	27.75 ± 5.08^*∗*^
IAD (*n* = 18)	7.67 ± 15.5^*∗*,*∗∗*^	312.06 ± 23.92^*∗*^	5,366.17 ± 1,434^*∗*^	308.92 ± 8.24^*∗*^	25.42 ± 1.38^*∗*^
CIP (*n* = 11)	2.81 ± 0.34	263.2 ± 23.85^*∗*^	3,800.36 ± 403^*∗*^	205.47 ± 97.63	21.19 ± 2.45

**Table 7:** MMP : TIMP ratios. *∗* shows significant differences to controls at *p* < 0.05. RAO: recurrent airway obstruction, IAD: inflammatory airway disease, and CIP: chronic interstitial pneumopathy.

	MMP-2 : TIMP-1	MMP-2 : TIMP-2	MMP-9 : TIMP-1	MMP-9 : TIMP-2	MMP-8 : TIMP-1	MMP-8 : TIMP-2
Controls (*n* = 15)	0.021	0.137	1.500	9.761	30.259	196.934
RAO (*n* = 17)	0.016	0.188	1.320	15.62^*∗*^	66.431^*∗*^	785.668^*∗*^
IAD (*n* = 18)	0.025	0.302^*∗*^	1.010	12.28	17.37^*∗*^	211.100
CIP (*n* = 11)	0.014	0.103	1.281	12.421	18.496^*∗*^	179.35
