# Corrigendum to “Metalloproteinases and Their Tissue Inhibitors in Comparison between Different Chronic Pneumopathies in the Horse”

**DOI:** 10.1155/2017/7825942

**Published:** 2017-12-11

**Authors:** Ann Kristin Barton, Tarek Shety, Angelika Bondzio, Ralf Einspanier, Heidrun Gehlen

**Affiliations:** ^1^Equine Clinic, Veterinary Faculty, Freie Universitaet Berlin, Oertzenweg 19b, 14163 Berlin, Germany; ^2^Animal Medicine Department, Faculty of Veterinary Medicine, Zagazig University, Zagazig, Egypt; ^3^Institute of Veterinary Biochemistry, Veterinary Faculty, Freie Universitaet Berlin, Oertzenweg 19b, 14163 Berlin, Germany

In the article titled “Metalloproteinases and Their Tissue Inhibitors in Comparison between Different Chronic Pneumopathies in the Horse” [1], there was a missing affiliation for the second author. The revised authors' list and affiliations are shown above.
